# Hand Eczema in the Polish Female Population

**DOI:** 10.3390/jcm12186102

**Published:** 2023-09-21

**Authors:** Agnieszka Polecka, Andrzej Awchimkow, Natalia Owsianko, Anna Baran, Justyna Magdalena Hermanowicz, Iwona Flisiak

**Affiliations:** 1 Department of Dermatology and Venerology, Medical University of Bialystok, Zurawia 14 St., 15-540 Bialystok, Poland; 2 Department of Pharmacodynamics, Medical University of Bialystok, Mickiewicza 2C St., 15-089 Bialystok, Poland

**Keywords:** hand eczema, eczema, disinfection, hand hygiene, female, skin, skin barrier

## Abstract

Background: This study aims to investigate the prevalence of hand eczema, its association with disinfectant usage during the COVID-19 pandemic, and potential correlations with age and dermatological history on hand symptoms in the Polish female population. Methods: A personalized online questionnaire was administered from January to March 2021 to 142 participants, including individuals with hand eczema. The questionnaire addressed demographics, dermatological history, disinfectant usage, and symptoms experienced during the pandemic. Results: The prevalence of hand eczema was higher in younger adults (aged 18–35), with significant exacerbations reported due to increased disinfectant usage. Respondents with a dermatological history were more susceptible to new skin symptoms during the pandemic. The quality of life was substantially impacted, particularly in individuals with hand skin dermatoses. Conclusions: The COVID-19 pandemic had a considerable influence on hand eczema, affecting prevalence, symptoms, and quality of life. Disinfectant usage emerged as a key factor in exacerbating hand skin lesions. Further research is warranted to explore the influence of specific disinfecting agents and improve treatment guidelines for personalized management of hand eczema. Despite limitations in the online survey method, these findings highlight the importance of proactive healthcare support for individuals with hand eczema during challenging times.

## 1. Introduction

Hand eczema (HE) is a common inflammatory, non-infectious skin condition affecting the hands, which significantly impacts the quality of life and functional abilities of those affected [1]. It is characterized by erythema, pruritus, edema, and occasionally the formation of painful vesicles or fissures on the skin. Except for its physical manifestations, the visible nature of the condition may lead to anxiety, depression, and social isolation. HE displays clinical heterogeneity encompassing diverse etiological factors, clinical phenotypes, and varying degrees of acuity. The etiology of this condition is multifaceted, stemming from endogenous sources, with atopic dermatitis as the most well-known endogenous risk factor, to exogenous triggers, including irritant and/or allergic contact dermatitis, which frequently exhibit overlapping manifestations [2,3,4]. HE can follow various time courses, including acute, recurrent, or chronic, with the potential for long-lasting and persistent manifestations. Prolonged exposure to irritants and allergens disrupts the skin’s natural barrier function, leading to an immune response and inflammation. The pathophysiology of hand eczema involves a complex interplay of immune-mediated reactions and the activation of inflammatory pathways. Approximately 5–8% of the general population experience HE, with a similar incidence rate observed in both adults and adolescents, while the one-year prevalence is estimated at 10%, with nearly one-third not seeking medical assistance for the disease [5]. Some studies suggest higher rates in specific occupational groups exposed to irritants and allergens. Additionally, regarding gender differences, hand eczema appeared to affect both men and women, with a higher 1-year prevalence observed among females than males, 11.5% vs. 6.7%, respectively. It is essential to note that the prevalence of hand eczema could be influenced by various factors, including changes in occupational practices, hygiene habits, exposure to irritants and allergens, and other environmental factors. Moreover, advancements in medical knowledge and increased awareness of hand eczema might impact the diagnosis and reporting of cases. During the COVID-19 pandemic, there was a widespread increase in hand hygiene practices and disinfectant usage as part of infection prevention measures. This surge in disinfection practices, including frequent hand sanitization, was crucial in reducing the transmission of the virus and protecting public health. Thus, it is essential to investigate the potential impact of increased hand hygiene and disinfectant usage on hand eczema in the population. The aim of the study was to investigate the relationship between hand eczema and increased disinfectant usage during the COVID-19 pandemic, as well as to provide evidence-based guidance for future public health responses and help to safeguard both individual skin health and overall public hygiene practices.

## 2. Materials and Methods

### 2.1. Ethical Approval and Informed Consent

This study was approved by the University Bioethics Committee (Ref.-No. APK.002.46 9.2020) and conducted in accordance with the Helsinki Declaration. All participants were informed about the conducted research and data collection. All respondents provided informed consent. Participation in the study was voluntary. Participants could withdraw their consent at any time during the survey.

### 2.2. Study Design and Population 

This was a descriptive, observational, original, online anonymous questionnaire survey evaluating the impact of disinfection on the condition of the skin of the hands and quality of life in the female population before and during the COVID-19 pandemic.

### 2.3. Study Protocol

This study was conducted in the form of a personalized, original, online questionnaire created by the authors of the study at the Department of Dermatology and Venereology, Medical University of Bialystok, Poland, from January to March 2021. The questionnaire was distributed electronically in Polish language via Google Forms through social media platforms (Facebook, WhatsApp) to the female population aged 18 years and older, including those with hand skin dermatoses. The survey consisted of 142 respondents and was designed to assess the impact of disinfection on hand skin conditions during the COVID-19 pandemic. The questionnaire was divided into four parts, assessing various aspects related to the participants’ demographics, dermatological history, current skin condition, treatment methods for hand skin lesions, and accompanying symptoms during disease exacerbations. The third part of the survey focused on identifying new symptoms or changes in the severity of hand skin lesions before and during the pandemic, particularly related to disinfection practices. It included questions on daily hand hygiene, frequency and volume of disinfectant usage, hand drying practices, and specific symptoms experienced after disinfectant application. Additionally, the survey collected data on changes in treatment approaches, frequency of dermatologist visits, remission periods during the pandemic, and alterations in skin moisturizing habits. 

To assess the impact of disinfection and quality of life during the pandemic, a 5-step visual analog scale was utilized, with 1 indicating no negative effects of disinfection and 5 denoting a significant impact on hand skin condition and a decline in quality of life. The survey questions underwent expert dermatologist review for consistency and appropriateness before implementation. The complete set of questions used in the survey can be found in Table S1 in the Supplementary Material.

### 2.4. Statistical Analysis

Data were collected in Excel, and statistical analyses were performed with the GraphPad Prism 9.20 software (GraphPad Software 225 Franklin Street. Fl. 26 Boston, MA 02110, USA). Descriptive analysis was performed by calculating the frequencies and percentages of variables. The relationships between the two variables were analyzed using the chi-square independence test. *p* values < 0.05 were considered statistically significant.

## 3. Results

### 3.1. Study Groups

**Figure 1:**
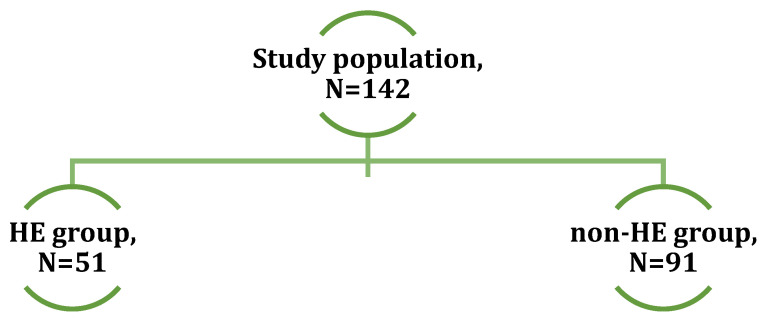
The division of study groups.

A total of 142 adult female individuals participated in an anonymous survey. Among the respondents, 51 subjects reported hand eczema diagnosed by a physician (HE group) (35.92%), including 8 participants (5.63%) who declared no dermatological symptoms before the outbreak of the pandemic. The second distinguished group, the non-HE group, consisted of individuals with no HE symptoms (controls) (Figure 1).

The demographic data of the study population are demonstrated in Table 1.

### 3.2. Study Outcomes

Study participants from the HE group were mainly aged between 18–24 (49.02%) and 25–35 (43.14%). Only 4 individuals were over 36 years old (7.84%). When focusing on the non-HE group, subjects were aged between 18–24 (60.44%) and 25–35 (35.16%). Four participants were older than 36 years old (4.40%) (Table 2).

**Figure 2:**
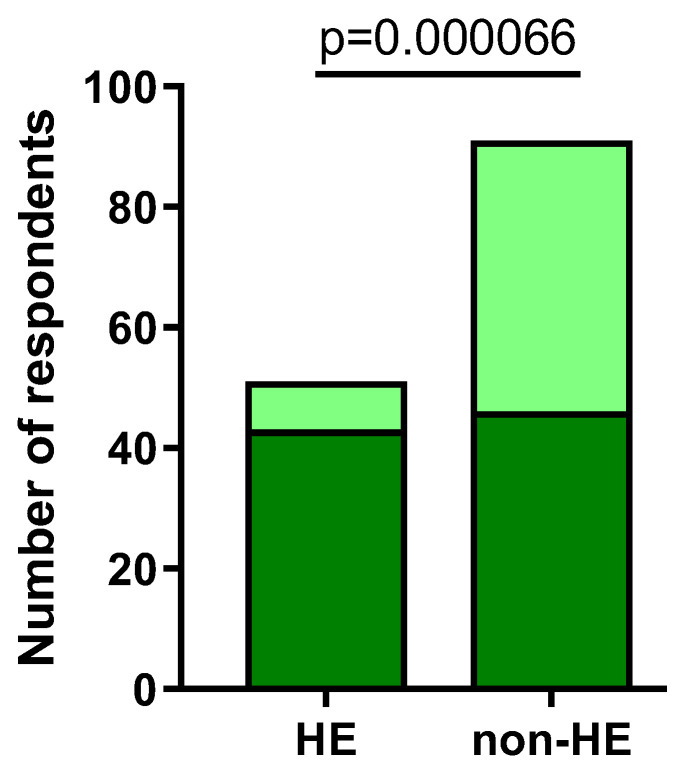
The number of respondents experiencing skin lesions worsening after subject-reported factors (dark green).

**Figure 3:**
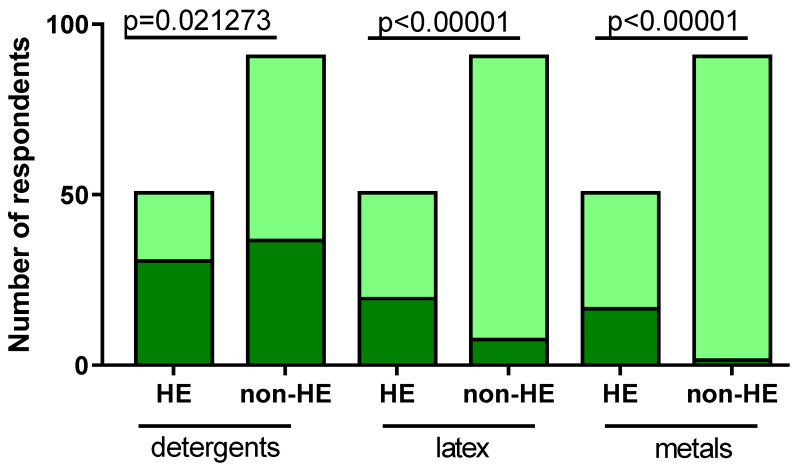
Factors aggravating skin lesions. dark green, subjects reported skin aggravation; light green, subjects with no skin aggravation.

Over 84% (43/51) of individuals from HE and 50% (46/91) subjects from the non-HE study group experienced worsening of skin lesion after the usage of subject-reported factors (*p* = 0.000066) (Figure 2). The factors that most frequently caused skin lesion exacerbation were detergents (in the HE group: 60.78% (31/51); in the non-HE group: 40.66% (37/91), *p* = 0.021273), latex (in the HE group: 39.22% (20/51); in the non-HE group: 8.79% (8/91), *p* < 0.00001), and metals (in the HE group: 33.33% (17/51); in the non-HE group: 2.20% (2/91), *p* < 0.00001) (Figure 3).

During increased hand skin alcohol-based disinfection in the prevention of coronavirus spread, 30/51 (58.82%) individuals of the HE group and 42/91 (46.15%) individuals from the non-HE group experienced worsening hand skin conditions (*p* = 0.147401). Mostly, the symptoms included: dryness (in the HE group: 21/30 (70%); in the non-HE group: 34/42 (80.95%)), roughness (in the HE group: 17/30 (56.67%); in the non-HE group: 30/42 (71.43%)), and redness (in the HE group: 15/30; 50%; in the non-HE group: 20/42 (47.62%)). Additionally, 20/91 individuals from the non-HE and 12/51 from the HE group reported the occurrence of new skin manifestations that had never been noticed before the COVID-19 pandemic (*p* = 0.831892). The new symptoms over the hand skin were: dryness (in the HE group: 7/20 (35%); in the non-HE group: 8/42 (19.05%)), roughness (in the HE group: 4/20 (20%); in the non-HE group: 4/42 (9.52%)), and redness (in the HE group: 1/20; 5%; in the non-HE group: 3/42 (7.14%)). Onychoschizia was reported in one non-HE subject and burning sensations in two non-HE individuals. Additionally, participants from the HE group noted symptoms that were not reported by subjects from the non-HE group: vesicles filled with serum fluid (2/20, 10%), and pustules (1/20, 5%), respectively. 

Respondents were allowed to select more than one symptom from those listed by the authors, and there was the option to include other conditions. Therefore, the results may exceed 100% of the total number of respondents from both study groups.

#### 3.2.1. Disinfection Habits

**Figure 4:**
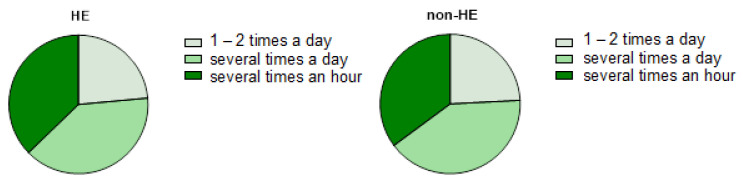
The frequency of skin disinfection in both groups.

In the HE-study group, 23.53% (n = 12/51) disinfected the skin about 1–2 times a day, 39.22% (n = 20/51) several times a day, and 37.25% (n = 19/51) disinfected several times an hour. Comparing, nearly ¼ of the non-HE group (24.18%, n = 22/91) disinfected their hands about 1–2 times a day, 40.66% (n = 37/91) several times a day, and 35.16% (n = 32/91) several times an hour (Figure 4). 

The vast majority of the HE (84.31%, 43/51) and non-HE (75.82%, 69/91) groups applied the appropriate, recommended by the manufacturer, volume of the disinfecting agents for the entire surface of the skin of the hands. The remaining respondents used more of the disinfectant than recommended by the manufacturer. None of the study participants indicated using too little of the product. 

Additionally, 8/51 of the HE and 8/91 of the non-HE group applied the disinfectant without hand drying.

#### 3.2.2. Symptoms after Disinfectant Usage 

**Figure 5:**
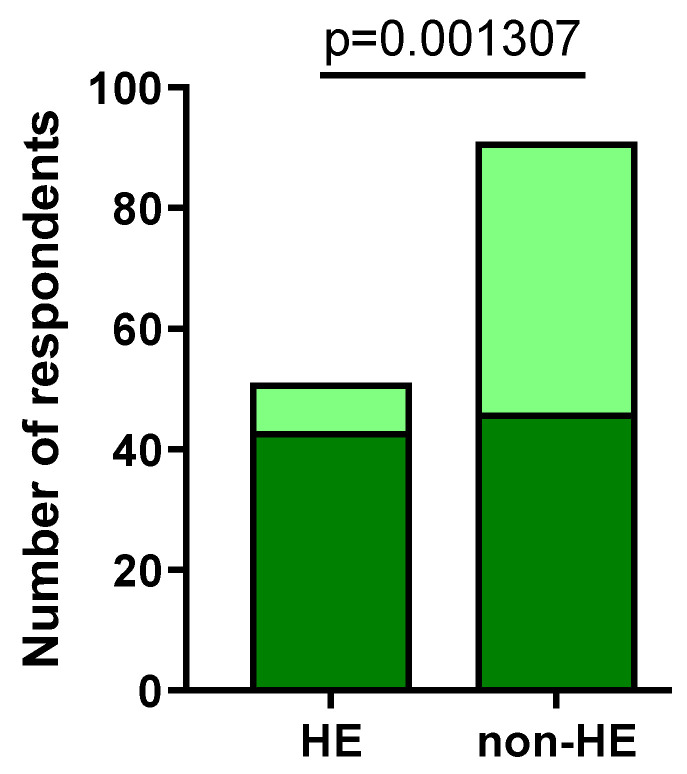
Number of respondents experiencing more than one skin symptom after disinfectant usage (dark green).

In total, 44/51 (86.27%) respondents from the HE group and 55/91 (60.44%) of the non-HE group experienced (immediately after the application of an alcohol-based disinfectant) more than one symptom (*p* = 0.001307), most often dryness (HE-study group: 43/44 (97.73%); non-HE group: 53/55 (96.36%)), roughness (HE-study group: 29/44 (65.91%); non-HE group: 41/55 (74.55%)), and redness (HE-study group: 31/44 (70.45%); non-HE group: 33/55 (60%)) (Figure 5).

**Figure 6:**
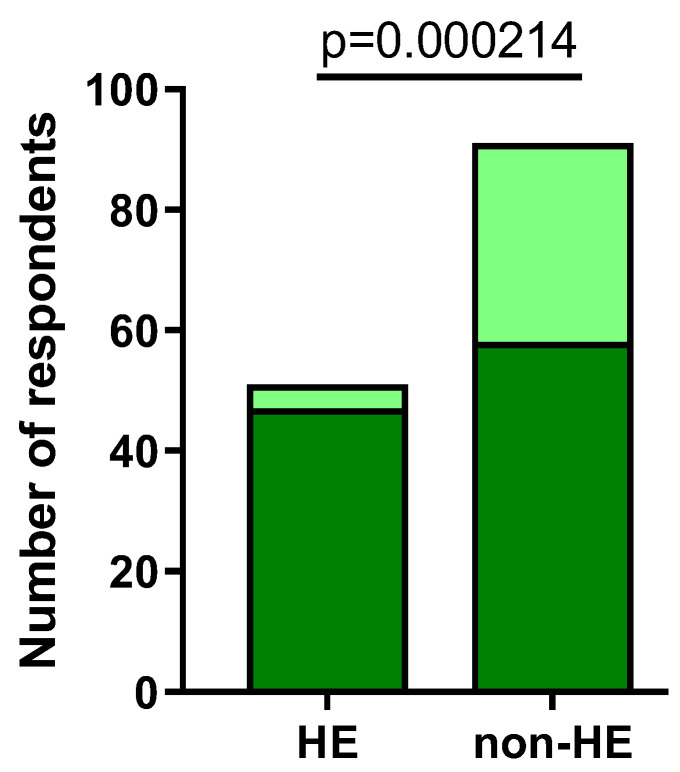
The number of respondents experiencing pain and burning sensations after disinfectant usage in both groups (dark green).

When asked only about pain and burning sensation (after the application of disinfectant agent), 47/51 (92.16%) respondents from the HE group and 58/91 (63.74%) from the non-HE group confirmed the appearance of the abovementioned symptoms (*p* = 0.000214) (Figure 6). 

Only 3/51 (5.88%) in the HE group and 20/91 (21.98%) in the non-HE group did not experience the negative symptoms of disinfection over the skin of the hands (*p* = 0.012504).

When analyzing pain and burning sensations after disinfectant usage correlated with the occurrence of new skin symptoms, the difference reached statistical significance in HE and non-HE study groups (*p* = 0.0239, and *p* = 0.0021, respectively). The presented data can be found in Figures S1 and S2 of the Supplementary Material.

#### 3.2.3. The Exacerbations of Skin Lesions

**Figure 7:**
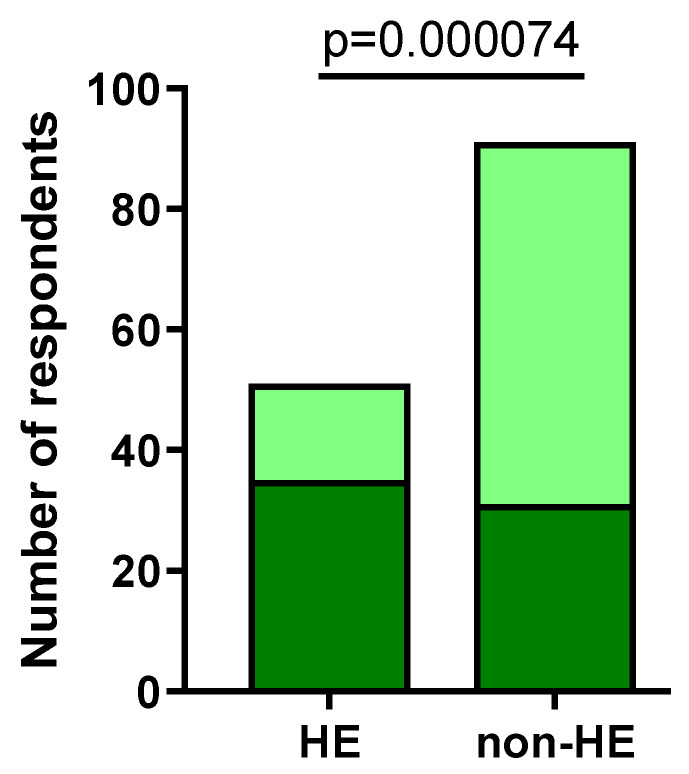
The number of respondents experiencing skin lesion exacerbations in both groups (dark green).

When comparing HE to the non-HE group, 35/51 (68.63%) and 31/91 (34.07%) participants experienced more exacerbations of the hand skin lesions after the pandemic outbreak (*p* = 0.000074) (Figure 7).

When analyzing exacerbations correlated with the frequency of skin disinfection, the difference reached statistical significance only in non-HE when comparing several times an hour to several times a day disinfection frequency (*p* = 0.023). The results obtained in the HE study group did not reach the significant difference (*p* = 0.075, and *p* = 0.0958, respectively). The presented data can be found in Figures S3 and S4 of the Supplementary Material.

#### 3.2.4. Medical Appointments

Due to skin changes since the start of using hand disinfection, participants from the HE group (14/51) had to seek medical advice significantly more often when compared to the non-HE group (3/91) (*p* = 0.000021). 

A total of 13/51 subjects from the HE group had to modify their pharmacotherapy and seek more advanced treatment methods (e.g., they were forced to switch from topical to systematic therapy, increase their medication dosage, or add a new drug). None of the subjects in the non-HE group had to change their treatment. 

When analyzing the correlation between the need to modify pharmacotherapy to more advanced treatment methods and the occurrence of new symptoms in the HE group, the difference did not reach statistical significance. The corresponding data are presented in Figure S5 of the Supplementary Material.

#### 3.2.5. Skin Infections

**Figure 8:**
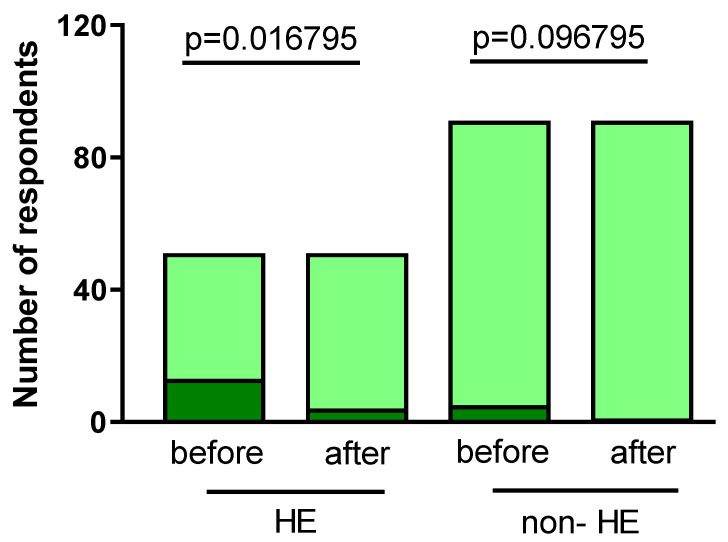
The number of respondents observing skin infections pre- and post-pandemic in both groups (dark green).

Before the outbreak of the pandemic, 13/51 of the HE group and 5/91 of the non-HE group observed skin infections over the hands. After increased disinfection related to COVID-19 prevention, the number of skin infections significantly decreased to 4/51 (*p* = 0.016795) in HE. There was a decrease observed in the non-HE group to 1/91 (*p* = 0.096795), but it was not significant (Figure 8).

#### 3.2.6. Skin Moisturizing

Before the pandemic, only 1/51 participants from the HE group did not moisturize the skin of the hands. In the non-HE group, there were 7/91 individuals who did not use emollients. After the COVID-19 pandemic, all subjects in the HE group moisturized the skin of the hands, while in non-HE, 5/91 declared no skin moisturization. Table 3 presents precise data on hand skin moisturization before and after the pandemic. 

#### 3.2.7. Quality of Life

**Figure 9:**
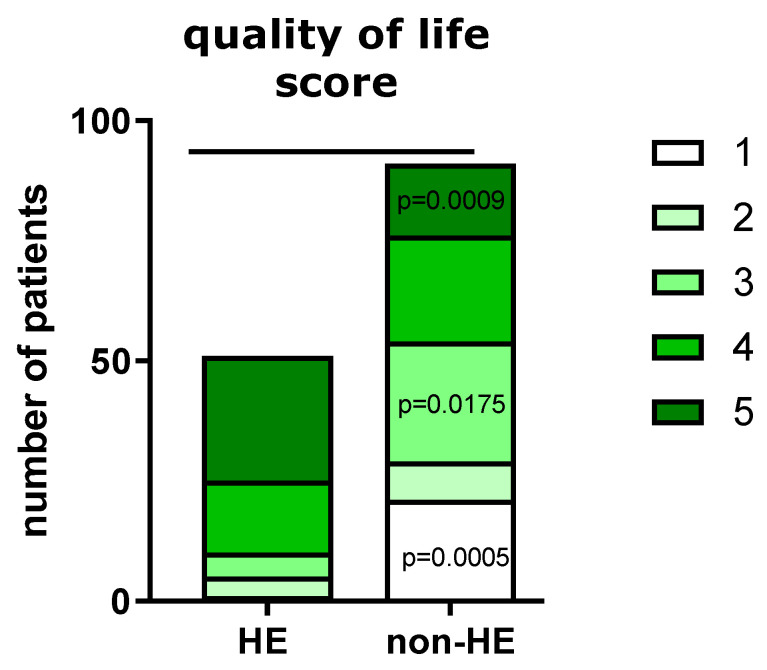
Answers distribution in both study groups.

To assess the quality of life (QoL), the 5-point visual analog scale was used. In the HE study group, 41/51 individuals indicated answers 4 and 5, while among the non-HE group, it was 37/91, respectively (*p* < 0.00001). When comparing the number of respondents in both groups declared QoL as 1, 3, and 5, there was a significant decrease in QoL in the HE group (Figure 9). The precise data summarizing the quality of study participants are presented in Table 4. 

## 4. Discussion

Hand eczema is a prevalent dermatological condition that can significantly impact the quality of life and skin health of affected individuals, particularly among the female population in Poland. HE affects women twice as often as men [6]. The gender disparity might be explained by women being more frequently exposed to activities involving wet work, such as cleaning, nursing, and hairdressing, for instance [7]. During the COVID-19 pandemic, increased hand disinfection practices became common to prevent the spread of the virus. This study explores the correlations between hand eczema and various subject-reported factors, as well as the impact of increased hand disinfection on hand skin conditions and quality of life in Polish females. 

In our study, the prevalence of HE is notable; it is estimated at 35.92% of study population. The data suggest that hand eczema might be a relatively common condition in the Polish female population. When comparing our results to the literature, the analysis by Quaade et al. on the general population estimated the overall pooled lifetime prevalence of HE to be 14.5% (95% CI: 12.6–16.5). All pooled prevalence estimates were higher among females than males, with a 1-year prevalence for females 11.5% (95% CI: 10.4–12.6) vs. 6.7% (95% CI: 5.8–7.8) for males. Likewise, all pooled prevalence estimates were higher among girls than boys [8]. Another study estimated the 1-year HE prevalence to nearly 10%, whereas the lifetime prevalence reached up to 20% [9]. Additionally, recent research found a statistically significant higher prevalence of hand eczema among women than men [10,11,12]. Moreover, our study revealed a striking urban predominance, with approximately 90% of the study population hailing from urban areas. This urban bias could be attributed to several factors. Cities typically offer improved infrastructure, including better access to healthcare facilities and a more comprehensive healthcare education. Moreover, the ease of internet accessibility in urban settings might have facilitated higher participation rates. However, it is important to acknowledge that this accessibility might have introduced a limitation to our findings, potentially excluding individuals with limited internet connectivity. When analyzing rural environments, 5 out of 15 participants were classified into the HE group, all of whom experienced new disinfection-related symptoms on the skin of their hands. Interestingly, among the rural participants in the non-HE group, 10 individuals denied the occurrence of any new skin symptoms. Agricultural and manual labor prevalent in rural areas can expose individuals to irritants and allergens that trigger and exacerbate the course of HE. Limited access to dermatological care in remote locations could lead to delayed diagnosis and treatment, additionally exacerbating the condition. Moreover, traditional practices involving prolonged exposure to water and detergents, common in rural settings, may contribute to HE development. Hence, considering the interplay between the advantages and risk factors of HE, further large-scale studies analyzing both rural and urban populations are necessary to enhance our comprehension of the prevalence landscape of hand eczema. 

Regarding the age distribution of participants in the HE group, the majority were in the younger age brackets, and only 7.84% of individuals were over the age of 36. Similarly, in the non-HE group, a significant number of participants were younger. Our data suggests that HE is more prevalent in younger individuals, particularly those aged between 18 and 35. This may be attributed to various factors such as increased exposure to wet work, irritants or allergens, higher rates of atopy, or genetic predisposition in younger age groups [9,13,14,15]. For instance, atopic dermatitis often starts in childhood and may persist into young adulthood. Approximately 25% of individuals who had moderate to severe atopic dermatitis during childhood will experience varying degrees of hand eczema in their adult lives [16]. Individuals with a history of atopic conditions, e.g., asthma, are more prone to developing HE [17]. Occupational factors and lifestyle may contribute to hand eczema development. Four research studies determined the mean age of onset for HE. In 2007, a cohort study conducted in Denmark revealed an overall mean age at onset of 24.3 years [18]. Similarly, another Danish study in the subsequent year reported a median age at onset of 27 years (26 years for females and 28 years for males) [19]. Additionally, a Swedish study from 2007 identified a notably lower mean age at onset (21.2 years) among 5034 females [20]. Furthermore, a Danish study from 2014, primarily focusing on young females (with a mean age of 22 years), reported a mean age at onset of 15 years [21]. Lastly, a recent Norwegian study documented a median age at onset of 22 years (21 years for females and 25 years for males) [22]. However, hand eczema can affect individuals of all ages. The relatively small proportion of individuals over the age of 36 in our study groups does not imply that HE is limited to younger age groups. The predominance of young females in online surveys might stem from various factors, such as their relatively easier access to the Internet and higher frequency of Internet usage. It is also possible that young females are more inclined to participate in online surveys due to factors such as their familiarity with technology, digital engagement, and willingness to share personal experiences. Moreover, the responsibility of managing household chores, including frequent cleaning and hand disinfection, might have contributed to their greater engagement in the study. This could be attributed to their roles in caregiving for children or elderly family members, which may have led them to seek information and solutions related to skin health. However, these factors collectively may have introduced selection bias, influencing the study’s generalizability and potentially limiting the representation of older individuals in the population. Further research is needed to better understand the nuances of participation patterns and their potential impacts on the prevalence of hand eczema.

The data analysis revealed significant correlations between specific subject-reported factors and the worsening of skin lesions in both the HE and non-HE study groups. The usage of detergents was found to be significantly associated with the exacerbation of skin lesions in both groups. Latex exposure also correlated with increased skin lesion exacerbation, and the incidence was notably higher in the HE group compared to the non-HE group. Additionally, exposure to metals showed a significant correlation with skin lesion exacerbation in the HE group but was rarely reported in the non-HE group. Metals, especially nickel, are well-known leading contact allergens in most industrialized countries worldwide, with a prevalence of up to 19% [23,24]. 

During the increased hand disinfection practices observed in the COVID-19 pandemic, a higher proportion of individuals from both the HE and non-HE groups experienced worsening hand skin conditions, although the difference was not statistically significant. The most frequently reported symptoms included dryness, roughness, and redness in both groups. Furthermore, some individuals from both groups reported new skin manifestations that were not present before the pandemic. However, there were no significant differences between the HE and non-HE groups in terms of new symptoms. The results correspond with the work of other investigators. Doğan et al. observed a significant increase in dryness, itching, pain, burning, erythema, and scaling in healthcare workers of COVID-19 and non-COVID-19 hospital units during the pandemic [25]. Babino et al. found the rising prevalence of irritant and allergic contact dermatitis in response to the COVID-19 pandemic [26]. It is noteworthy that participants from the HE group reported specific symptoms, such as vesicles filled with serum fluid and pustules, which were not reported by subjects from the non-HE group. Conversely, onychoschizia and burning sensations were reported only by non-HE individuals.

The data revealed interesting patterns in hand disinfection practices among the HE and non-HE study groups. Both groups exhibited a high frequency of hand disinfection, with a considerable proportion disinfecting their hands multiple times a day. However, there were no significant differences between the two groups in terms of the frequency of disinfection. Furthermore, a vast majority of participants in both the HE and non-HE groups applied the recommended volume of disinfectant for the entire surface of their hands, as recommended by the manufacturer. This indicates that most individuals were following proper hand disinfection protocols, which is crucial in the context of infection prevention, particularly during the COVID-19 pandemic. However, it is noteworthy that a subset of respondents in both groups used more disinfectant than recommended by the manufacturer. This may have implications for skin health, as excessive use of disinfectants can lead to skin dryness and irritation. Additionally, a small number of participants in both groups applied the disinfectant without proper hand drying. This practice can further exacerbate skin irritation and may have contributed to the reported symptoms in both groups. 

In the context of hand eczema, the impact of disinfectant use on skin health is critical, with disinfectants potentially exacerbating symptoms, particularly when inadequately dried. Healthcare professionals should guide proper hand disinfection, especially for those with pre-existing hand skin conditions, as these are at increased risk of developing new symptoms during the pandemic [27,28]. Our study highlights a significant discrepancy in symptoms post-alcohol-based disinfectant application between HE and non-HE groups. HE respondents reported multiple symptoms (e.g., 3 and more) compared to non-HE (e.g., 1 or 2 symptoms), encompassing dryness, roughness, redness, pain, and burning, echoing Techasatian et al. [28]. Notably, a larger percentage of the HE group experienced these symptoms compared to the non-HE group. Interestingly, a small subset in both groups did not encounter negative symptoms. Nonetheless, the HE group showed significantly fewer symptom-free individuals (5.88%) compared to the non-HE group (21.98%). This underscores that hand eczema has higher susceptibility to adverse skin reactions from alcohol-based disinfectants. Notably, symptoms such as dryness, roughness, redness, pain, and burning were more prevalent in the HE group. Such results underscore the necessity of considering the effects of alcohol-based disinfectant on hand skin health, especially in pre-existing HE cases. Furthermore, a statistically significant correlation was observed between pain and burning sensations after disinfectant usage and the occurrence of new skin symptoms within both the HE and non-HE groups. This implies a shared impact of intensified disinfection practices on skin health. Notably, irrespective of the presence of hand eczema, participants who developed new symptoms on their hand reported experiencing consistent pain and burning sensations, indicative of discomfort linked to these skin manifestations. Disinfection is known to compromise the skin’s hydrolipid barrier [29,30], and the heightened exposure during the pandemic may amplify symptom susceptibility. Deeper penetration of disinfectants into the epidermis and dermis likely contributes to post-disinfection pain and burning sensations. While a correlation exists between new symptoms and the frequency of pain and burning sensations among those experiencing skin symptoms, we observed an elevated occurrence of pain and burning in the HE group. This could be attributed to the inflammation and hyperreactivity characteristics of hand eczema, rendering the skin more susceptible to external irritants [31]. 

The study data revealed a significant difference in the frequency of exacerbations of hand skin lesions between the HE and non-HE study groups. A higher proportion of participants in the HE group reported experiencing exacerbations of hand skin lesions after the pandemic outbreak compared to the non-HE group. This significant difference underscores the impact of the pandemic on individuals with hand eczema, leading to more frequent and pronounced exacerbations in this population. Interestingly, irrespective of the frequency of disinfection usage among HE participants, the risk of exacerbation remained consistent. In contrast, within the non-HE groups, only participants who employed disinfection several times per hour exhibited an elevated risk of developing exacerbations. In light of these findings, providing appropriate patient information regarding the need to reduce disinfectant usage could serve as a crucial preventive measure to mitigate the risk of exacerbations. When considering the higher rate of exacerbations in the HE vs. non-H group, this may be attributed to various factors. During the pandemic, individuals were exposed to increased stress, anxiety, and changes in daily routines, which could have contributed to the worsening of hand eczema symptoms in susceptible individuals. The stress resulting from a novel situation and sudden changes in daily routines could play a role in aggravating and precipitating new skin conditions [32]. Furthermore, the pandemic’s everyday life was characterized by isolation and anxiety about the well-being of loved ones, as reported by Lewicka et al., which has been associated with increased stress and anxiety levels [33]. The existing literature indicates that specific stressors have been linked to more frequent exacerbations and a deteriorating course of disease in patients with atopic dermatitis [34,35]. Stress is a well-established trigger for atopic dermatitis and can disrupt the proper functioning of the skin’s epidermal barrier through mechanisms involving the stress-induced secretion of endogenous glucocorticoids, potentially leading to damage to the skin barrier [36,37].

Poorly treated skin lesions worsen the course of skin dermatoses [38]. The participants from the HE group sought medical advice significantly more frequently than those from the non-HE group due to skin changes since the start of using hand disinfection. This suggests that hand disinfection practices during the pandemic may have exacerbated hand eczema symptoms in susceptible individuals, leading to a higher demand for medical assistance. The increased need for medical advice in the HE group may be attributed to the skin’s heightened sensitivity and susceptibility to irritants, such as alcohol-based disinfectants, which are commonly used during infectious disease outbreaks. Moreover, a considerable proportion of individuals from the HE group had to modify their pharmacotherapy to more advanced treatment methods, such as transitioning from topical to systematic therapy, increasing medication dosage, or adding new drugs to their treatment regimen. Despite the occurrence of new symptoms within the HE group, the analysis revealed no significant difference in the modification of pharmacotherapy to more advanced treatment methods. In contrast, none of the participants from the non-HE group required changes in their treatment. This suggests that hand-skin symptoms in the HE group may have become more severe or refractory, necessitating more aggressive therapeutic interventions. The observed differences in medical management between the HE and non-HE groups highlight the impact of hand eczema exacerbations on the healthcare system and the increased burden on medical resources. Individuals with hand eczema experiencing worsened symptoms due to hand disinfection practices may require more frequent medical visits, specialist consultations, and adjustments to their treatment plans, putting additional strain on healthcare facilities. These findings underscore the importance of considering hand eczema management in the context of infectious disease outbreaks when hand hygiene practices, including frequent hand disinfection, become critical for infection control. Healthcare professionals should be aware of the potential impact of hand disinfection on hand eczema and be prepared to provide appropriate guidance and support to individuals with hand eczema during such challenging times.

More than half of all HE patients are colonized by bacteria (*Staphylococcus aureus*, *S. aureus*), and the risk of colonization is strongly related to the severity of the disease [39,40]. Ong et al. and Haslund et al. reported that individuals with hand skin dermatoses with impaired skin protective barriers and immune processes face a higher susceptibility to microbial infections, particularly bacterial (*S. aureus*) and viral infections [40,41]. Before the outbreak of the pandemic, a higher proportion of individuals in the HE group (13/51) reported experiencing skin infections over the hands compared to the non-HE group (5/91). This finding suggests that individuals with hand eczema may be more susceptible to developing skin infections due to the compromised skin barrier and the presence of inflammation in hand eczema [29,30]. However, an interesting observation emerged after the increased hand disinfection practices related to COVID-19 prevention. In the HE group, the number of skin infections significantly decreased to 4/51, indicating a notable reduction in the occurrence of skin infections among individuals with hand eczema. This decrease can be attributed to the heightened emphasis on hand hygiene practices during the pandemic, which may have contributed to better infection prevention and reduced exposure to external pathogens. In the non-HE group, a decrease in the number of skin infections was also observed, but it did not reach statistical significance. This suggests that while increased hand hygiene practices may have had a positive impact on reducing the incidence of skin infections in the general population, they may not have had as pronounced an effect on individuals without hand eczema.

However, it is crucial to consider the specific needs of individuals with hand eczema, as they may still be at a higher risk of skin infections despite increased hand hygiene measures. The compromised skin barrier and increased vulnerability to irritants and allergens in hand eczema can predispose individuals to infections, even with improved hand hygiene practices. These findings underscore the importance of providing tailored guidance and support to individuals with hand eczema to maintain optimal hand skin health during periods of increased hand disinfection. Education on the proper use of disinfectants, frequent moisturization, and avoidance of potential triggers can help mitigate the risk of exacerbations and skin infections in this population.

Emollients are topical formulations composed of vehicle-type substances without active ingredients and are used to create a protective barrier on the skin [42]. They help to retain moisture and protect the skin from irritants [43]. During the pandemic, the application of skin moisturizers was primarily geared towards therapeutic use rather than preventive measures, as emollients were introduced only when respondents started encountering new skin conditions [44]. Before the pandemic, the majority of participants in both the HE and non-HE groups reported regular hand skin moisturization. We found a high adherence to hand moisturization practices in the HE group. Similarly, in the non-HE group, a small percentage of participants reported not using emollients, suggesting that the majority of individuals in this group also practiced hand skin moisturization. Interestingly, after the COVID-19 pandemic, there was a notable change in hand skin moisturization practices in both study groups. In the HE group, all subjects reported moisturizing the skin of their hands, indicating a universal adoption of hand moisturization practices among individuals with hand eczema during the pandemic. This shift may be attributed to the increased emphasis on hand hygiene practices and the awareness of the potential drying effects of frequent hand disinfection, prompting individuals with hand eczema to prioritize hand skin moisturization. In the non-HE group, a small proportion of participants reported not using emollients after the pandemic. However, the majority of individuals in this group continued to practice hand skin moisturization, with similar distribution across several times a week, 1–2 times a day, and >3 times a day. The results suggest that individuals in both study groups recognized the importance of hand skin moisturization, particularly during the pandemic when frequent hand disinfection practices were implemented to prevent viral transmission. Hand skin moisturization is crucial for maintaining the skin barrier function and preventing excessive dryness and irritation, especially in individuals with hand eczema, who may already have a compromised skin barrier. The high adherence to hand skin moisturization practices observed in both the HE and non-HE groups demonstrates the awareness and proactive approach of the participants toward hand health during challenging times. The universal adoption of hand moisturization in the HE group may have contributed to better hand skin health and potentially mitigated exacerbations of hand eczema symptoms during the pandemic. Moreover, public health initiatives and educational campaigns should focus on promoting alternative hand hygiene practices, such as the use of moisturizing soaps and gentle cleansers, to mitigate the adverse effects of frequent hand disinfection on hand skin health. 

The pandemic has deeply influenced the well-being of individuals, impacting various facets of their lives. The heightened emphasis on hygiene and exacerbated skin issues contributed to a considerable decline in the overall quality of life, particularly among respondents with hand skin dermatoses. To assess QoL, a 5-point visual analog scale was utilized, with higher scores indicating a more negative impact on QoL. The mean QoL in the HE group was observed to be significantly higher compared to the non-HE group. This finding indicates that individuals with hand eczema experienced a more significant decrease in their QoL during the pandemic, likely attributed to the exacerbation of hand eczema symptoms due to increased hand disinfection practices. The data further revealed that a higher proportion of individuals in the HE group (41/51) indicated answers 4 and 5 on the QoL scale, reflecting a substantial negative impact on their QoL due to hand disinfection. In comparison, a smaller percentage of individuals in the non-HE group (37/91) reported answers 4 and 5 on the QoL scale, suggesting a lesser impact of hand disinfection on their QoL. The significant decrease in QoL in the HE group compared to the non-HE group highlights the considerable burden of hand eczema and its exacerbation during the pandemic. The findings emphasize the importance of considering the psychological and emotional aspects of hand eczema management, especially HE, which can have a profound impact on the individual’s self-esteem, social interactions, and overall psychological well-being. Healthcare professionals should be aware of the potential impact of hand disinfection practices on the QoL of individuals with hand eczema and provide appropriate support and interventions to address the psychosocial aspects of the condition. Providing psychological support, patient education, and counseling on coping strategies can play a crucial role in improving the QoL of individuals with hand eczema and promoting overall well-being during challenging times. Interestingly, the individual scores evaluating QoL in patients in accordance with the skin region affected by the disease should be considered. Corazza et al. conducted a study on eczematous individuals with 43.36% face and 56.64% hand involvement. PRISM and DLQI scores showed a moderate to strong inverse correlation, but PRISM revealed a higher sensitivity in capturing patients’ suffering than DLQI, especially in the case of face involvement. Itching was the sole parameter significantly associated with both PRISM and DLQI scores. PRISM appeared to be more accurate in detecting the burden of eczematous diseases involving the face, probably due to the interception of the emotional impact, while DLQI, focusing on patient functioning, was more affected by hand involvement. Site involvement could be a criterion for selecting the best QoL assessment tool [45]. 

Nevertheless, it is important to acknowledge the limitations of our study, which primarily stem from the online surveying method, which relied on respondents’ subjective perspectives. Individuals who choose to participate in an online questionnaire may not represent the entire population of interest, leading to self-selection bias. Those with a specific interest or experience related to the topic may be more likely to respond. As researchers, we were unable to physically examine the condition of the respondents’ hands. Moreover, the survey’s accessibility was restricted to individuals with Internet and social media access, potentially excluding some segments of the population. Additionally, it is worth noting that older individuals who may be less familiar with or have limited access to the Internet could introduce a potential bias in the study’s findings. Moreover, the study’s demographic analysis lacks consideration of manual labor or work involving social interactions, and these factors hold significance in comprehensively defining the impact on QoL in HE. Further studies are needed to conduct a thorough and accurate assessment of the prevalence of HE, ensuring a robust and comprehensive understanding of this dermatological condition.

## 5. Conclusions

In conclusion, our study sheds light on the impact of the COVID-19 pandemic on hand eczema in the Polish female population. The prevalence of hand eczema was notably higher in younger adults. Despite the study limitations, the data obtained from the literature corresponds with our results. We observed a significant correlation between hand eczema and the increased use of alcohol-based disinfectants during the pandemic, leading to various symptoms such as dryness, roughness, and redness. Moreover, respondents with a dermatological history were at a heightened risk of developing new skin symptoms during the pandemic, necessitating more frequent visits to dermatology practices. The pandemic’s profound effects on daily life, along with the emphasis on hygiene practices and stressors, likely contributed to the exacerbation of hand skin lesions, resulting in a significant reduction in the overall quality of life, particularly in individuals with hand skin dermatoses. Looking forward, further research is warranted to explore the influence of specific disinfecting agents on the development and exacerbation of hand skin lesions. Efforts should be made to improve treatment guidelines tailored to the unique needs of individuals with hand eczema, considering personalized medicine approaches.

## Figures and Tables

**Table 1:** Demographic characteristics of the study population.

Characteristics	N (%)
**Gender**	
Female	142 (100%)
**Age (years)**	
18–25	80 (56.34%)
26–35	54 (38.03%)
36–45	3 (2.11%)
>45	5 (3.52%)
**Education**	
Primary	2 (1.41%)
Secondary	73 (51.41%)
Higher	67 (47.18%)
**Residence**	
Rural	15 (10.56%)
City to 250,000 inhabitants	47 (33.10%)
City with more than 250,000 inhabitants	80 (56.34%)

**Table 2:** The age structure in the study groups.

Age (Years)	HE Group, N (%)	Non-HE Group, N (%)
18–24	25 (49.02%)	55 (60.44%)
25–35	22 (43.14%)	32 (35.16%)
36–45	2 (3.92%)	1 (1.10%)
>45	2 (3.92%)	3 (3.30%)

**Table 3:** The frequency of hand skin moisturization in study groups.

	Pre-Pandemic	Post-Pandemic	*p* Value
	**HE**	**HE**	
several times a week	10	9	
1–2 times a day	20	14	* *p* < 0.001
>3 times a day	20	28	
	**non-HE**	**non-HE**	
several times a week	41	20	
1–2 times a day	31	37	** *p* < 0.01
>3 times a day	12	27	

*—measured as HE pre-pandemic several times a week/sum of pre-pandemic 1–2 times a day and >3 times a day/HE post-pandemic several times a week/sum of post-pandemic 1–2 times a day and >3 times a day, **—measured as non-HE pre-pandemic several times a week/sum of pre-pandemic 1–2 times a day and >3 times a day/ non-HE post-pandemic several times a week/sum of post-pandemic 1–2 times a day and >3 times a day.

**Table 4:** Assessment of the quality of life associated with hand disinfection in both groups during the pandemic.

Answer	HE Group, N (%)	Non-HE Group, N (%)
1	1 (1.96%)	21 (23.08%)
2	4 (7.84%)	8 (8.79%)
3	5 (9.80%)	25 (27.42%)
4	15 (29.41%)	22 (24.18%)
5	26 (50.98%)	15 (16.48%)

1—disinfection had no negative effect on the quality of life, 5—disinfection had a significant impact by lowering quality of life.

## Data Availability

Not applicable.

## Supplementary Materials

The following supporting information can be downloaded at: https://www.mdpi.com/article/10.3390/jcm12186102/s1, Table S1: The questions included in the survey. Figure S1. The correlation between pain and burning sensations after disinfectant usage and the occurrence of new skin symptoms in the HE study group. Figure S2. The correlation between pain and burning sensations after disinfectant usage and the occurrence of new skin symptoms in the non-HE study group. Figure S3. The correlation between exacerbations and the frequency of skin disinfection in the non-HE study group. Figure S4. The correlation between exacerbations and the frequency of skin disinfection in the HE study group. Figure S5. The correlation between the need to modify pharmacotherapy to more advanced treatment methods and the occurrence of new symptoms in the HE group.
