# Supplementary material for: Hand Eczema in the Polish Female Population
Source: J Clin Med. 2023 Sep 21;12(18):6102. doi: 10.3390/jcm12186102 (PMC10531572; doi:10.3390/jcm12186102)
Supplement: Supplementary file 1 [file jcm-12-06102-s001.zip › jcm-2577871-supplementary.pdf]

Survey questions
1. Gender<br>2. Age<br>3. Education and/or profession<br>4. Residence
1. Have you ever suffered from hand skin diseases in the past, and if so, what was the diagnosis?<br>2. Do you currently suffer from hand skin diseases, and if so, what is the diagnosis?<br>3. Have you had patch tests/biopsy to confirm hand skin disease? What allergens are you allergic to? What did the biopsy reveal?<br>4. If you have not had patch tests performed or biopsy, do you notice deterioration of the skin condition after any factors, and if so, which ones?<br>5. Do you use dermatological treatment, and if so, what kind?<br>6. What hand skin symptoms did you experience during exacerbations of the diseases?<br>7. Have you noticed any new skin symptoms over the hands while using disinfectants, and if so, which ones?
1. How often during the day do you use hand disinfection?<br>2. What volume of hand disinfectant do you use during each application?<br>3. Do you dry your hands thoroughly before applying hand disinfection?<br>4. Do you experience any pain or burning sensations during hand disinfectant application?<br>5. Do you experience any negative symptoms after applying the disinfectant?<br>6. Since you started using hand disinfection in accordance to the SARS-CoV-2 pandemic, have you noticed any new hand skin symptoms that were never noted before, and if so, which ones?<br>7. Have you noticed more frequent exacerbations of hand skin lesions since you started using hand disinfection, and if so, which ones?<br>8. Since you started using hand disinfection during the pandemic, have you had to use medications to

alleviate skin symptoms more frequently, if so, which ones?

9. Since the start of the use of hand disinfection, have you noticed any changes in time needed to achieve a state of remission of the disease?
10. Since the start of the use of hand disinfection, have you had to seek more frequent medical advice/visits due to hand skin lesions?
11. Since you started using hand disinfection, have you had to change treatment method to control skin symptoms?
12. Has the duration of remission (symptom-free period) during the pandemic significantly changed compared to the period before hand sanitizer use?
13. Before the period of use of hand disinfectants, did you ever have microbial hand superinfections (bacterial, viral, fungal), and if so, which ones?
14. Before the pandemic, had you been tested for bacterial and fungal hand skin superinfections, and if so, what was the result of the test?
15. Have you had microbial skin superinfections during the pandemic, and if so, which kind of superinfection?
16. Have you been tested for bacterial and fungal superinfections of hand skin during the pandemic, and if so, what was the result of the test?
17. Did you use moisturizing products - emollients (e.g. creams, lotions, milks, ointments) - before the pandemic, and if so, how often?
18. Did you use moisturizers, emollients during the pandemic, and if so, how often?
19. When have you used moisturizers, emollients (e.g. before/after hand washing, before/after hand disinfection, when experienced dryness)?
20. Using a scale of 1 to 5, how would you rate the nuisance of using disinfection if 1 means disinfection had no negative effect on the quality of life, 5 – a significant impact of disinfection on the lowering quality of life?

Table S1. – The questions included in the survey.

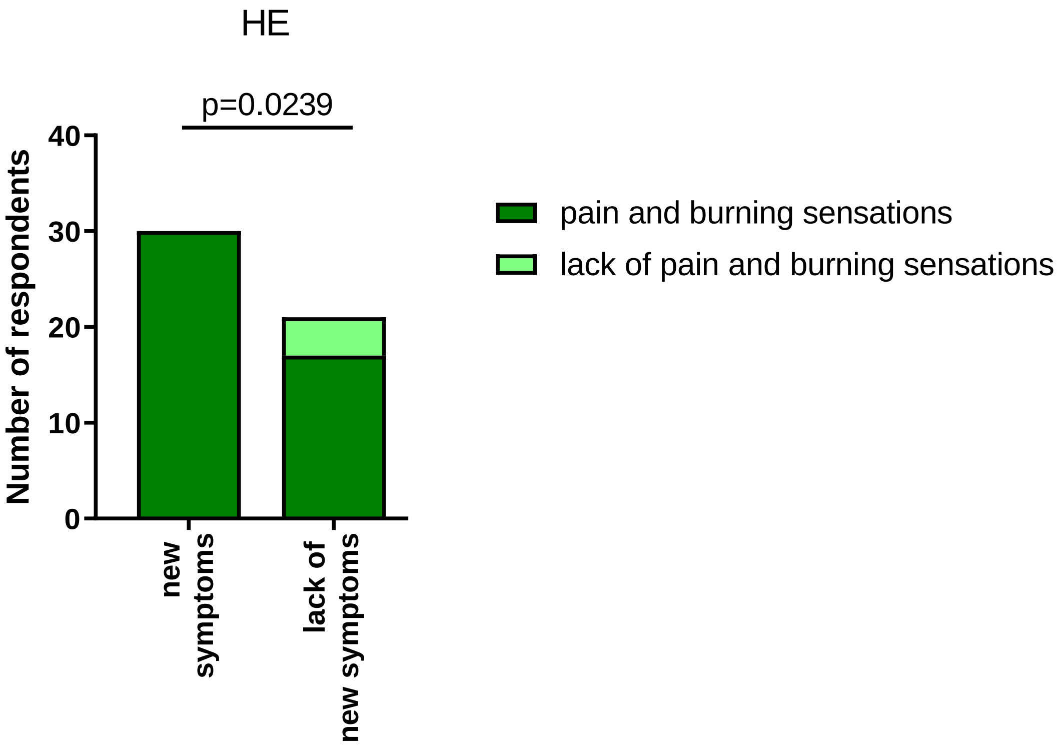

Figure S1. The correlation between pain and burning sensations after disinfectant usage and the occurrence of new skin symptoms in the HE study group.

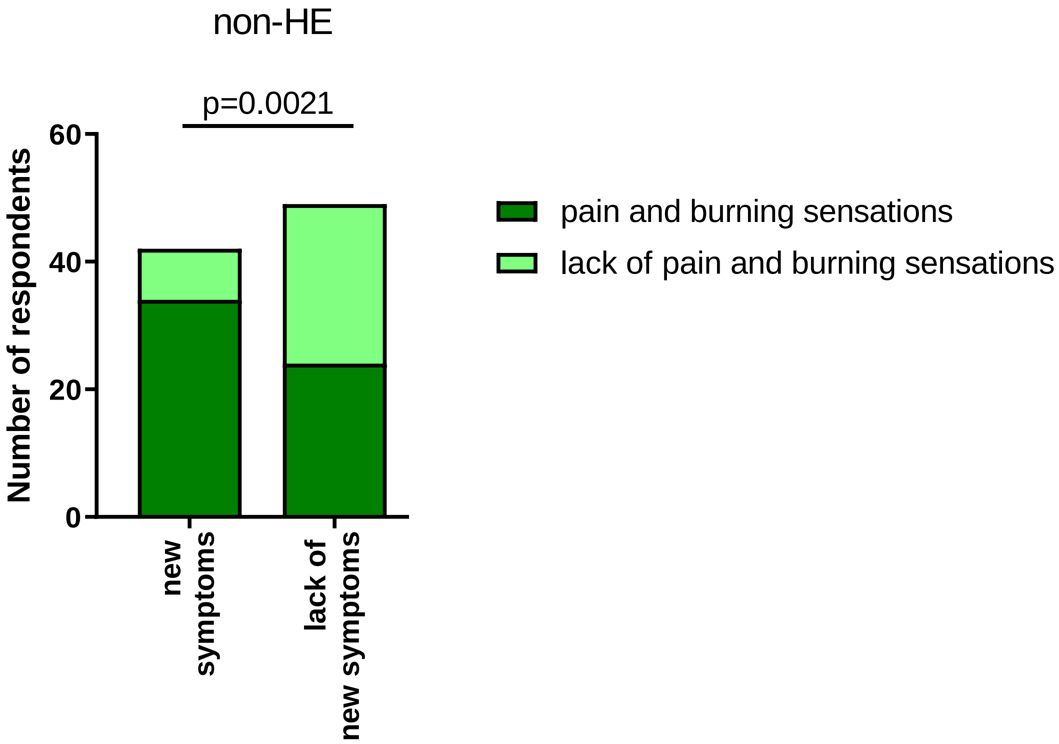

Figure S2. The correlation between pain and burning sensations after disinfectant usage and the occurrence of new skin symptoms in the non-HE study group.

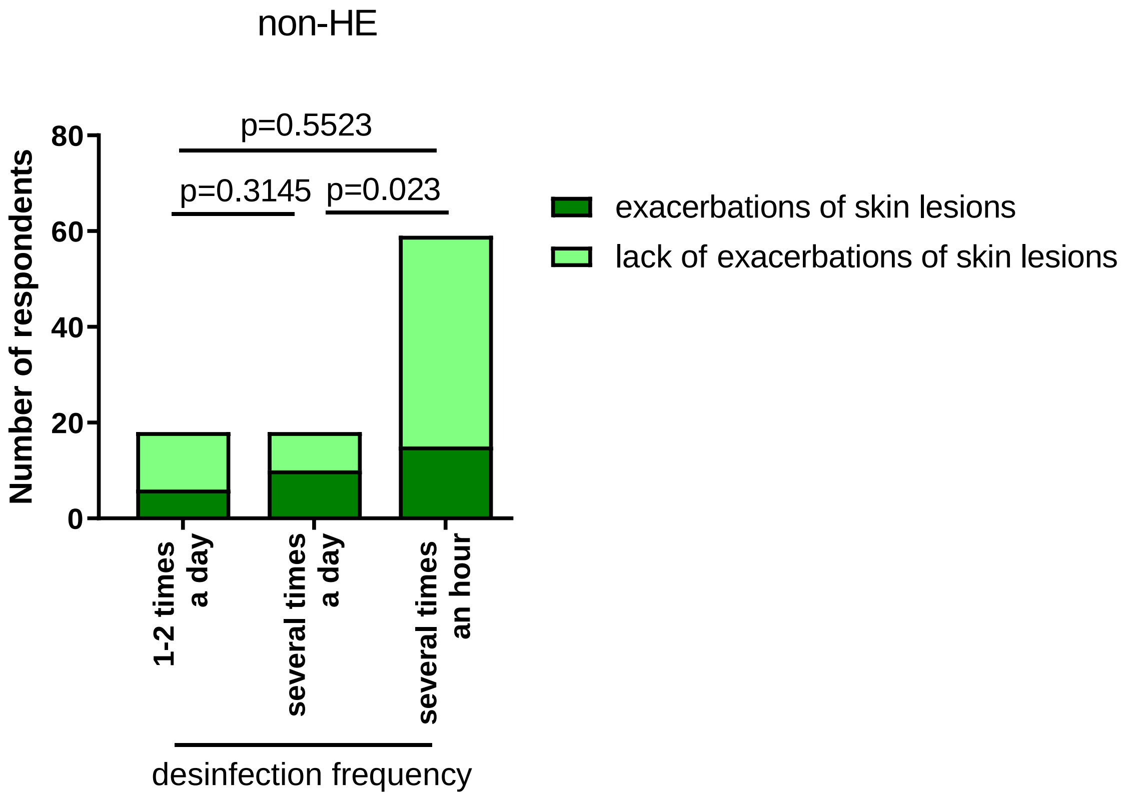

Figure S3. The correlation between exacerbations and the frequency of skin disinfection in the non-HE study group.

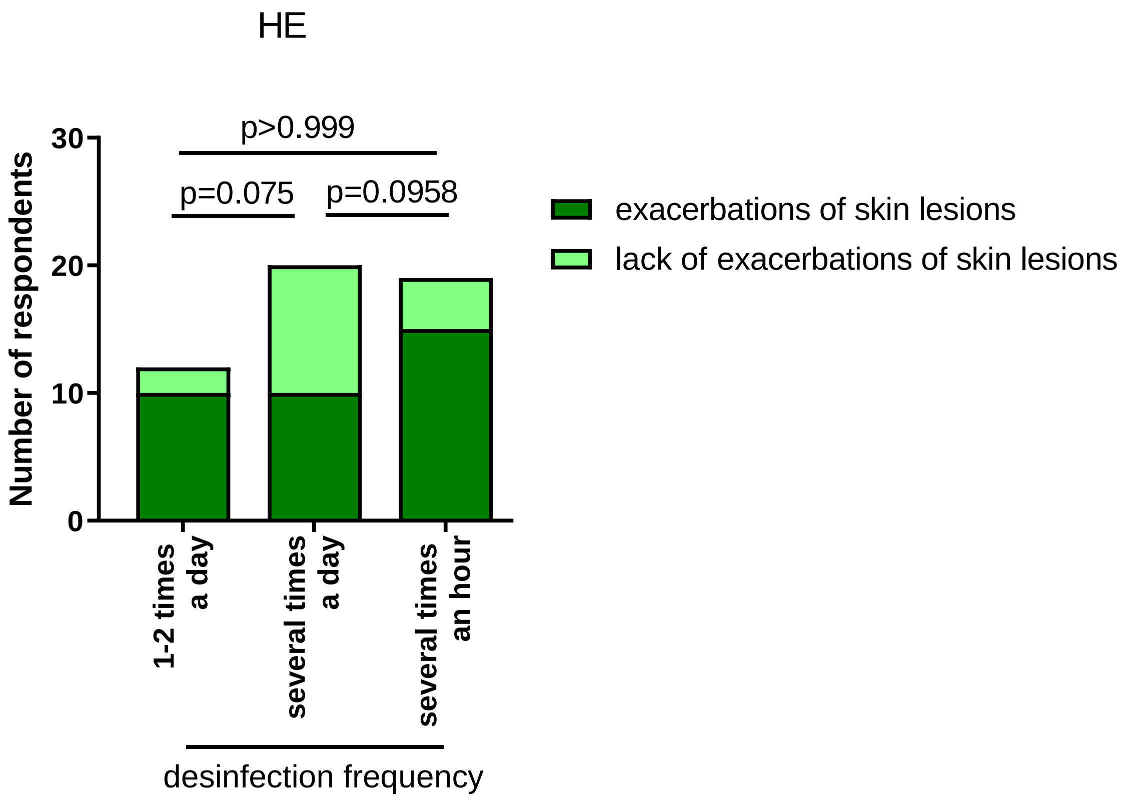

Figure S4. The correlation between exacerbations and the frequency of skin disinfection in the HE study group.

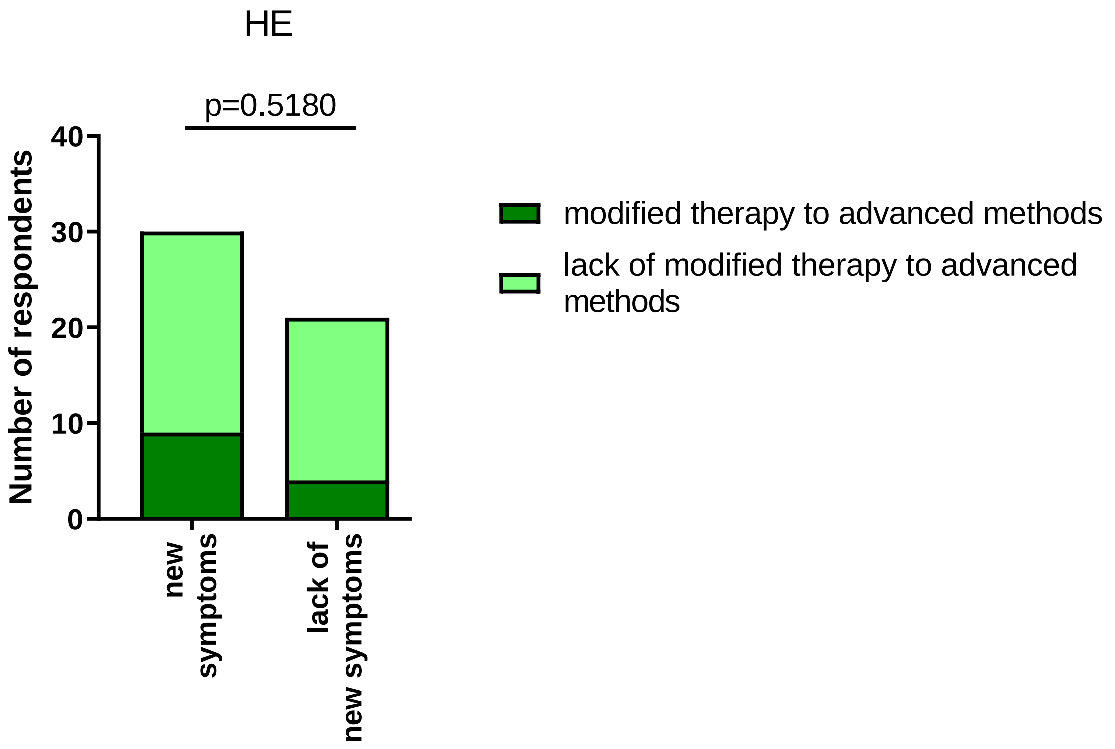

Figure S5. The correlation between the need to modify pharmacotherapy to more advanced treatment methods and the occurrence of new symptoms in the HE group.
